# A Novel Quantitative Approach for Eliminating Sample-To-Sample Variation Using a Hue Saturation Value Analysis Program

**DOI:** 10.1371/journal.pone.0089627

**Published:** 2014-03-03

**Authors:** Katsumi Yabusaki, Tyler Faits, Eri McMullen, Jose Luiz Figueiredo, Masanori Aikawa, Elena Aikawa

**Affiliations:** 1 Center for Interdisciplinary Cardiovascular Sciences, Division of Cardiovascular Medicine, Brigham and Women's Hospital, Harvard Medical School, Boston, Massachusetts, United States of America; 2 Center for Excellence in Vascular Biology, Division of Cardiovascular Medicine, Brigham and Women's Hospital, Harvard Medical School, Boston, Massachusetts, United States of America; National Cancer Institute, National Institutes of Health, United States of America

## Abstract

**Objectives:**

As computing technology and image analysis techniques have advanced, the practice of histology has grown from a purely qualitative method to one that is highly quantified. Current image analysis software is imprecise and prone to wide variation due to common artifacts and histological limitations. In order to minimize the impact of these artifacts, a more robust method for quantitative image analysis is required.

**Methods and Results:**

Here we present a novel image analysis software, based on the hue saturation value color space, to be applied to a wide variety of histological stains and tissue types. By using hue, saturation, and value variables instead of the more common red, green, and blue variables, our software offers some distinct advantages over other commercially available programs. We tested the program by analyzing several common histological stains, performed on tissue sections that ranged from 4 µm to 10 µm in thickness, using both a red green blue color space and a hue saturation value color space.

**Conclusion:**

We demonstrated that our new software is a simple method for quantitative analysis of histological sections, which is highly robust to variations in section thickness, sectioning artifacts, and stain quality, eliminating sample-to-sample variation.

## Introduction

For over a century, histological analysis of biological samples has led to greater understanding of biological mechanisms. The ability of researchers to interpret the data present in histological images has been the limiting factor to the usefulness and power of histology and histopathology. Histological assessment is often used as a qualitative method by clinical pathologists and within research settings, localizing a specific biomarker in the tissue or exploring tissue morphology and remodeling. Qualitative histological analyses have contributed importantly to our knowledge of cellular and tissue anatomy. The well-known Golgi method elucidated the structure of the nervous system at the turn of the 20th century, and by combining advanced fluorescent stains with time-lapse photography, modern researchers can track the migration of individual sub-cellular structures such as mitochondria [1] or matrix vesicles [2]. Qualitative analyses remain useful for diagnosing disease; frozen section biopsies are commonly used to identify cancers, and analysis of cultures can help identify bacterial species. However, as methods of immunohistochemical staining have advanced, histological diagnoses and research have become more refined. Instead of merely testing for the presence or absence of a biomarker, experimental pathologists and histologists began utilizing semiquantitative techniques [3]. The most common form of such analysis in histology requires that a researcher create a rubric for assigning a score to each experimental tissue sample. These scores may rely on a histologist's experience and intuition and could be imprecise or subjective, and difficult to recreate exactly [4].

In order to affix frozen tissue samples to slides, histologists use cryotomes, specialized devices that can slice frozen samples into sections only a few microns thick. Cryotomes can be adjusted to cut sections to a range of thicknesses as necessary. Certain tissues or histological stains may call for sections as thin as 2 µm, while others may require sections greater than 20 µm thick. However, cryotomes are imperfect machines. Studies have shown that standard laboratory cryotomes may produce sections that vary in thickness by 11% on average [5], or in the best case scenario sections may have a coefficient of variation of 3.3% [6]. Individual cryostats may be more or less precise, depending on manufacturing quality, age, level of maintenance, and cutting temperature. Similar concerns can be applied to microtomes specialized to section paraffin-embedded tissues.

While variation in section thickness is relatively small, it can impact quantitative image analysis by changing the color properties of light transmitted through the tissue sample. Thicker sections will bind more stain (increasing the saturation) and will increase in opacity (decreasing the value and intensity of the sample). The impact of these variations can be lessened by staining replicate sections, but this may be difficult in experiments where sections are hard to obtain, or a limited amount of sectionable tissue is available. Resolving these problems requires new methods for quantification of histological images that do not depend on tissue thickness or observer expertise.

The availability of recent digital image analysis software has enabled fully quantitative, observer independent histological analyses. Instead of assigning arbitrary scores to stained samples, researchers can use computers to assess each pixel of a digital image and determine whether it displays stain character or not, and calculate the percent area that displays the target stain. Despite its usefulness, the quality of the digital photograph and the method used to identify positive stain limit the power of this technique. In addition, tissue section thickness variation still impairs the results. We therefore developed method, utilizing hue saturation value (HSV) color space, which provides accurate quantitative data independent of observer biases and section thickness.

A “color space” is any mathematical representation of perceived colors. Most modern computer monitors physically represent color in red-green-blue (RGB) space: each pixel of a computer screen emits narrowband light at three frequencies: red, green, and blue. In standard color digital photography, an image is stored as information describing an ordered collection of pixels, each of which can be defined by its location in the image and its position in the RGB color space. However, in a standard color digital image, each pixel can instead be defined by three other characteristics: hue is related to the dominant wavelength displayed by the pixel; saturation is a basic measure of the purity of the hue (or apparent grayness of the pixel); value is a measure of the intensity of light shining from the pixel. Each of these characteristics has a finite range of possible values, and therefore the entire color space can be represented by a 3-D graph with its three axes representing the three characteristics. Points within this graph that lie near each other represent colors that are very similar to each other; points that are extremely close together may represent colors that are indistinguishable to the naked eye. Here we present a novel program, *Color Selection HSV* (*CSH*), which uses the HSV color space to analyze histological images.

## Methods

To test our program, *CSH*, we used it to perform color separation and positive area selection on various sample images. We obtained all of the experimental images for this study using a Nikon Eclipse 50i light microscope and a Nikon Digital Sight DS Fi1c digital camera. We compared the output from *CSH* to the output from a conventional RGB-based image analysis program.

Our program, *Color Selection HSV*, is available for use as a web application at http://cics.bwh.harvard.edu. It accepts most image files as an input, including .jpg, .png, .tiff, .gif, and .bmp. Users can set up to three sets of thresholding parameters, allowing three distinct color ranges to be extracted from an image.

### Color separation

We used two color spaces, the RGB color space and the HSV color space, to separate colors in our experimental images. The RGB color space has 3 color dimensions, comprising red (R), green (G), and blue (B), and any digitally recorded color can be expressed by a unique combination of these 3 values. Almost all digital imaging devices, including digital cameras and image scanners, use the RGB color space to store colors in image files as numerical values. The HSV color space also has 3 color dimensions, comprising hue (H), saturation (S), and value (V). The principal wavelength expressed by a color is entirely represented by hue. Saturation specifies how pure or gray a color is, and value specifies the brightness of the color. Because the vast majority of electronic images are stored as RGB values, we performed a mathematical conversion from the RGB color space to the HSV color space. Conversions from RGB to HSV are unique, repeatable, and reversible. The detailed explanation for our specific color conversion formulae can be found in Methods S1, along with some examples in Table S1.

Using these two color spaces, we prepared two software tools, *Color Selection HSV*, which converts image data to the HSV color space, and a similar program which leaves images in the RGB color space for analysis. These tools automatically extract certain colors from images by locating every pixel which falls within specified threshold ranges in each of the three dimensions of the relevant color space. We designed the tools to be able to extract three different colors from an image by preparing three independent sets of threshold ranges within each of the color dimensions. We show how the thresholding procedure works in Figure S1, and the appearance of the graphical user interface of our software is shown in Figure S2.

### Histological approach

All tissues used to test our program were mouse tissues frozen in Optimal Cutting Temperature (OCT) compound. Tissue was harvested from animal experiments approved by the Beth Israel Deaconess Medical Center Institutional Animal Care and Use Committee, Protocols # 010-2013, 048-2010, and 017-2010. Unless otherwise specified, samples were cut to 6 µm slices, and were fixed with 10% neutral buffered formalin for 5 minutes immediately prior to staining.

Masson's Trichrome (collagen accumulation detection): Sample slides were immersed in Bouin's fluid for 30 minutes, then washed thoroughly in tap water. Samples were then immersed in Weigert's Hematoxylin solution for 5 minutes before being washed in tap water again, then placed in Biebrich scarlet-acid fuchsin solution for 10 minutes. After another round of tap water washing, samples were moved into phosphotungstic-phosphomolybdic acid for 30 seconds, washed, and stained with aniline blue for 5 minutes. Slides were then dehydrated in progressive alcohol washes, and placed in xylene before coverslipping.

MAC3 Immunohistochemistry (macrophage detection): Sample sections were fixed in cold acetone (−30°C) for 5 minutes in lieu of formalin, then allowed to dry at room temperature for 15 minutes. Slides were placed in 0.3% hydrogen peroxide for 20 minutes, washed in tap water, then placed in phosphate buffered saline (PBS). Sections were then incubated at room temperature for 30 minutes with 4% normal rabbit serum. Anti-mouse MAC3 antibody was applied to each section at a dilution of 1∶900, and sections were allowed to incubate for 90 minutes. Sections were washed in PBS, and incubated with a rabbit anti-rat secondary antibody for 45 minutes. Sections were then washed in PBS and incubated with horseradish derived peroxidase streptavidin for 20 minutes and washed again. Sections were developed using a 3-amino-9-ethylcarbazole (AEC) substrate-chromagen development solution from Dako. Nuclear staining was achieved using Gill's hematoxylin followed by ammonium water. The slides were coverslipped using an aqueous mounting medium.

Picrosirius Red (fibrillar collagen detection): Sample slides were immersed in picrosirius red solution (0.1 g Sirius red per 100 mL picric acid) for 90 minutes, then washed in 0.01 N hydrochloric acid twice. Sections were dehydrated using progressive alcohol washes, placed in xylene, coverslipped, and visualized using a polarizing light microscope (Nikon eclipse 80i).

Alkaline Phosphatase (ALP): Reagents from Vector Labs' Vector Red Alkaline Phosphatase Substrate Kit I were mixed with 5 mL of 100 mM Tris-hydrochloride and used to cover sample sections for 60 minutes. Slides were rinsed first in 100 mM Tris-hydrochloride, then in water. To achieve nuclear counterstaining, slides were dipped in Gill's hematoxylin for 5 seconds, rinsed in water, and submerged in ammonium water for 1 minute. Slides were rinsed with water, dehydrated using progressive alcohol washes, placed in xylene, and coverslipped.

### Statistical analysis

Statistics was performed using *t* test. Data are presented as mean ± SEM. *P* values less than 0.05 were considered significant.

## Results

### Color selection HSV maps images to the HSV color space

**Figure 1:**
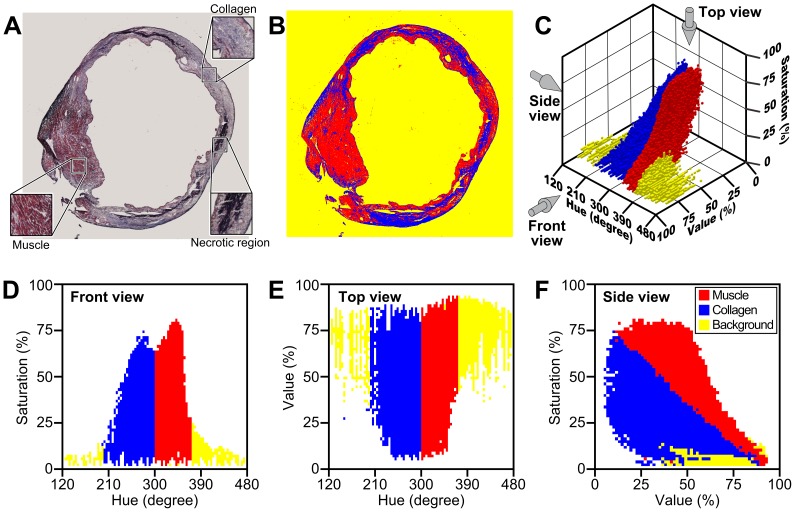
*CSH* processing on an infarcted mouse heart. A) An infarcted mouse heart section cut 8 µm thick. Muscle tissue is scarlet red, while collagen fibers appear blue, and necrotic regions are purple-black. Insets show enlarged areas of muscle, collagen and necrotic region. B) The same mouse heart, post-processing by *CSH*. The areas that *CSH* determined as collagen are blue, and the areas that *CSH* determined as muscle are red. The background is yellow. C) A plot of the pixels from the original heart image mapped to HSV space. The gray arrows indicate the direction from which this 3-D graph will be displayed in the following 2-D images. D) A plot of the pixels from the original image in the Hue-Saturation plane. The borders collagen and the muscle rectangular thresholds are visible at Hue = {200, 300, 385}. E) A plot of the pixels from the original image in the Hue-Value plane. F) A plot of the pixels from the original image in the Value-Saturation plane. This graph most clearly shows the different shapes of the collagen peak (blue) and the muscle peak (red).

Figure 1 shows the analysis process *CSH* performed on an infarcted mouse heart stained with Masson's trichrome method. *CSH* mapped each pixel in the original image (Figure 1 A) to the HSV space using the mathematical equivalency described in Methods S1 and Table S1. *CSH* then generated two thresholding cuboids within the HSV space in order to identify pixels that are of similar color to one another and are of experimental interest; in this case, pixels marked as red represent muscle tissue while pixels marked as blue represent collagen fibers. *CSH* displays the original input image as well as the modified image, where pixels of interest have been recolored (Figure 1B). Figure 1 shows the mathematical mapping of the original image, including a 3-D plot of HSV space (Figure 1C) as well as the maps looking down the Value axis (Figure 1D), the Saturation axis (Figure 1E) and the Hue axis (Figure 1F). Selected pixels can be redefined to new, user-assigned color values in order to be easily visually identified. *CSH* also counts the total number of pixels that fall into each threshold and displays this number as both a scalar and as a percentage of the region of interest (ROI).

### *CSH* shows consistent measurements of collagen accumulation independent of section thicknesses, magnification, pixel size, artifacts, and image focus

**Figure 2:**
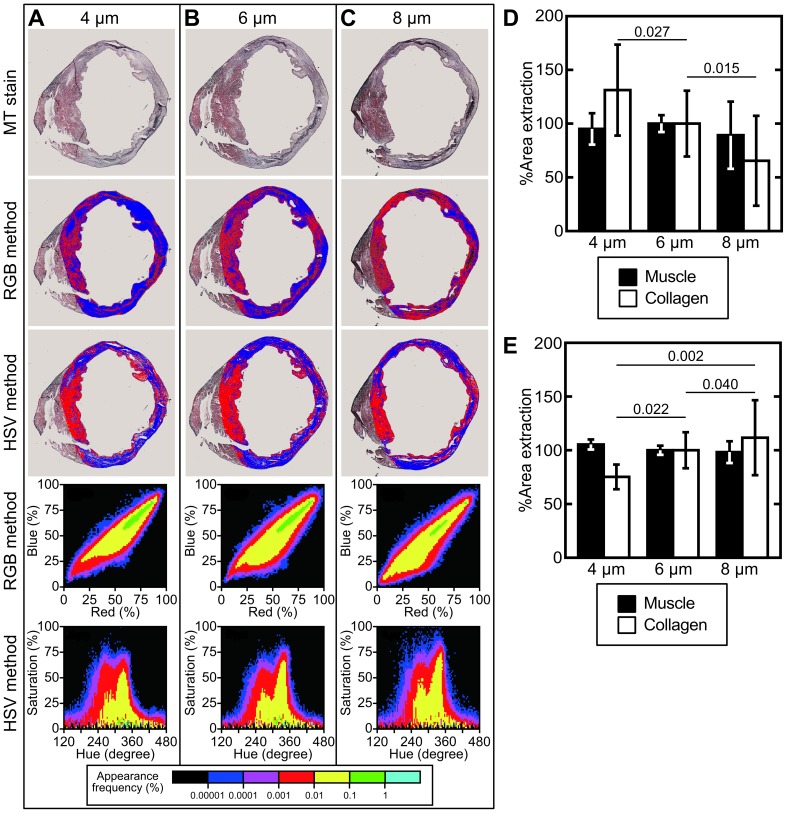
Analytic performance across diverse section thicknesses. A) A section of infarcted mouse heart cut to 4 µm and stained with Masson's trichrome. Descending from the original image, we see the RGB binary image, the HSV binary image, a density map of the pixels mapped to the RGB color space, and a density map of the pixels mapped to the HSV color space. B) A section of infarcted mouse heart cut to 6 µm and stained with Masson's trichrome. C) A section of infarcted mouse heart cut to 8 µm and stained with Masson's trichrome. D) For each of four experimental hearts and each of the three section thicknesses, the area identified as muscle is plotted next to the area identified as collagen using the RGB method. Because each heart has a different size infarction, these results for each heart are normalized as a percentage of the measured area in the 6 µm sample. As the section thickness increases, RGB analysis decreases the perceived collagen area, despite analyzing adjacent sections of heart. E) For each of four experimental hearts and each of the three section thicknesses, the area identified as muscle is plotted next to the area identified as collagen using the HSV method. Because each heart has a different size infarction, these results for each heart are normalized as a percentage of the measured area in the 6 µm sample. There is no discernible change in perceived muscle or collagen area as the section thickness increases when using the HSV method.

In order to test the analytic power and consistency of *CSH*, we analyzed adjacent tissue samples that differed only in thickness. Four mouse hearts, each with significant myocardial infarction, were embedded in OCT compound and frozen. Using a cryotome, triplicate sections of the hearts were cut to 4 µm, 6 µm, and 8 µm (Figures 2 A–C). As seen in the first row of Figure 2, these sections were stained with Masson's trichrome simultaneously and under identical conditions. Digital images were taken of these sections using a 2× scanning power objective lens. We performed image quantification analysis on these photographs using both a classic RGB-thresholding method as shown in the second row of Figure 2, and our new HSV-thresholding method as seen in the third row of Figure 2. We optimized the thresholding parameters for each method using a single image, then left those parameters constant for all subsequent analyses. The RGB analysis method returned inconsistently diverse areas, which were identified as collagen vs. muscle as section thickness changed (Figure 2D). The HSV method showed more consistency within each section thicknesses (Figure 2E).

Next, we analyzed *CSH*'s ability to accurately process images at various magnifications. We took digital images of regions of MI mouse hearts, stained with Masson's trichrome, which included the border between muscle tissue and collagen, using 2×, 10×, and 20× objective lenses (Figure S3). We cropped the lower magnification images so that they each featured the same heart portion. We analyzed these images with both the RGB method and *CSH*. The RGB method provided drastically different results depending on magnification, while *CSH* remained constant across all magnifications.

Similarly, we tested *CSH*'s ability to withstand decreased image resolution. To accomplish this, we took a representative MI mouse heart image (3000×3000 = 9 million pixels), taken with a 2× objective lens, and serially decreased its size, in pixels, by one-half in each dimension (Figure S4). *CSH* only began returning inconsistent results once the image was reduced to 375×375 = 140,625 pixels, while the RGB method provided inconsistent results with images as large as 750×750 = 562,500 pixels.

Certain common histological artifacts, such as ripples, tears, and folds, can impede quantification of images. We tested *CSH'*s ability to overcome even large folds in tissue sections. We examined a portion of an MI mouse heart section with a large fold which passed through both muscle and collagen areas (Figure S5). *CSH* was able to accurately discern muscle tissue from collagen, even in the folded portion of the section. The RGB method was unable to do that; instead it excluded the entirety of the fold from analysis.

Occasionally, due to imperfections in sectioning, slides, or imaging, portions of a sample image may be out of focus. We tested *CSH*, as well as an RGB method of analysis, on a partially defocused image by tilting a sample slide by 6°, and taking multiple photographs of it with a shifting focal plane (Figure S6). *CSH* provided highly conserved analyses between focal planes, whereas the RGB method struggled to consistently measure collagen area.

### *CSH* provides measurements of detailed features

**Figure 3:**
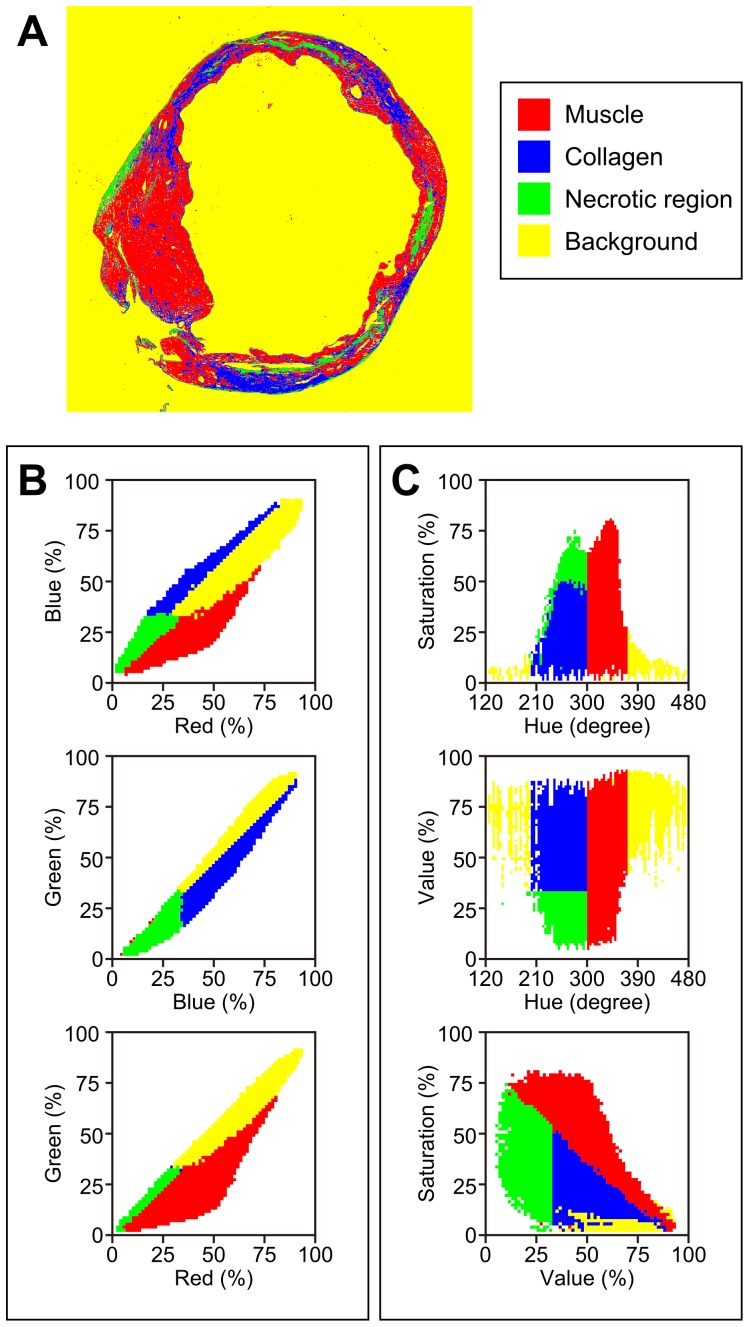
Additional thresholds are possible with *CSH*. An infarcted mouse heart, analyzed by *CSH* for collagen, muscle, and necrotic tissue. A) A post-processed image of an infarcted mouse heart using the HSV method that includes a binary for necrotic tissue. B) 2-D plots of the original pixels mapped to the RGB color space, but with the same binary applied as in Figure 3A. C) 2-D plots of the original pixels mapped to the HSV color space.

To further display the potential advantages of *CSH*, we set a third thresholding cuboid in order to identify necrotic regions, in addition to muscle and collagen, within our mouse hearts (Figure 3A). Given the narrow, fusiform shape of the color distribution of the hearts in RGB space, it is difficult to set an accurate threshold for the dark necrotic tissue (Figure 3B). By transforming the color data into HSV space (Figure 3C), the shape of the color map changes and spreads out, revealing distinct clusters of pixels that correspond to the different tissue types present in the section. Dark necrotic regions can therefore be easily identified by the software, yielding more detailed and useful information about the section.

### *CSH* is suitable for accurate quantitative immunohistochemistry

**Figure 4:**
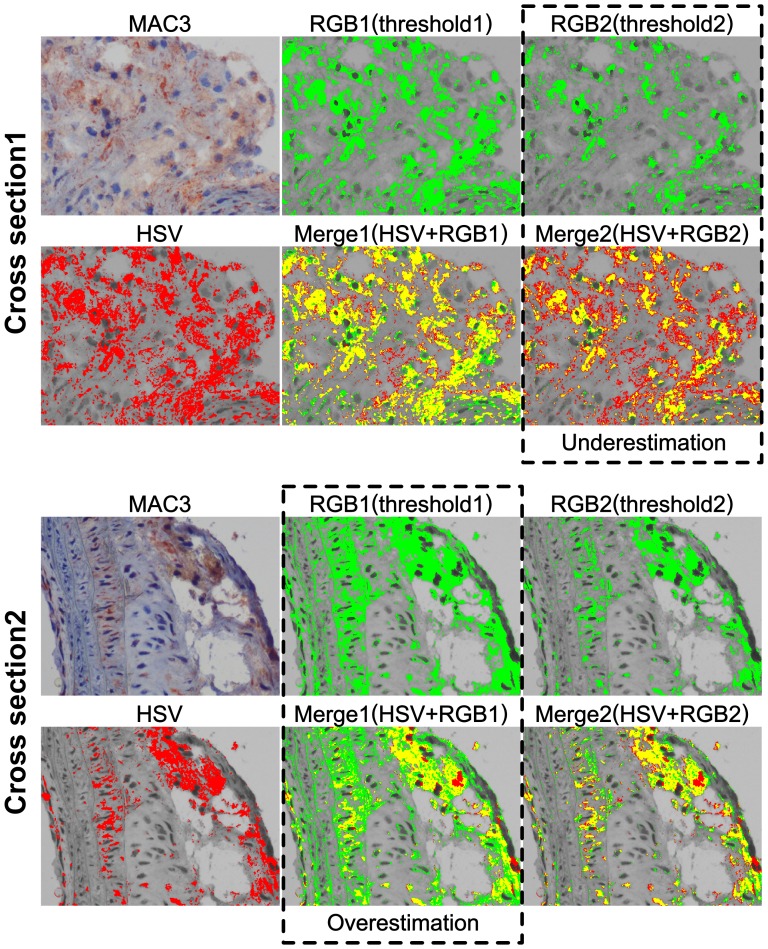
*CSH* is consistent between individuals. Apoe−/− mouse innominate arteries stained with MAC3 antibody for detection of macrophages. RGB1(threshold1) was optimized for Cross Section 1, and overestimates the positive area when applied to Cross Section 2. RGB2(threshold2) was optimized for Cross Section 2, and underestimates the positive area when applied to Cross Section 1. *CSH* was able to effectively use a single HSV threshold on both cross sections. In the overlays between the HSV and RGB1 and RGB2, yellow area shows where there is agreement between the HSV method and the RGB method. Green area in the overlays may indicate false positive area reported by the RGB method, while red area in the overlays may represent false negative area reported by the RGB method.

The advantages *CSH* offers over RGB methods are not restricted to sections of variable thickness. *CSH* also provides greater consistency and power whenever individual samples display a wide variety of stain intensity. To show this, we performed immunohistochemistry on the innominate arteries of two ApoE−/− mice, using MAC3 (macrophage detection) as a primary antibody (Figure 4). We labeled HSV signal as red and RGB as green, and overlap between the two shown as yellow. The plaque present in cross section 1 displays a more spread out and less intense signal than the plaque from cross section 2, making it hard to set useful thresholds for RGB analysis. When the RGB threshold was optimized for cross section 1, the program falsely identified large areas from cross section 2 as positive, as shown in the cross section 2 panel labeled “Merge1 (HSV+RGB1)” (green shows overestimation). Conversely, when the threshold was optimized for cross section 2, large portions of positive signal were falsely identified as negative in cross section 1, as shown in the cross section 1 panel labeled “Merge 2 (HSV+RGB2)” (red shows underestimation). Using *CSH*, when the threshold was optimized for cross section 1, it remained accurate and appropriate for the cross section 2 as well.

### *CSH* is suitable for accurate quantification of specialized stains

**Figure 5:**
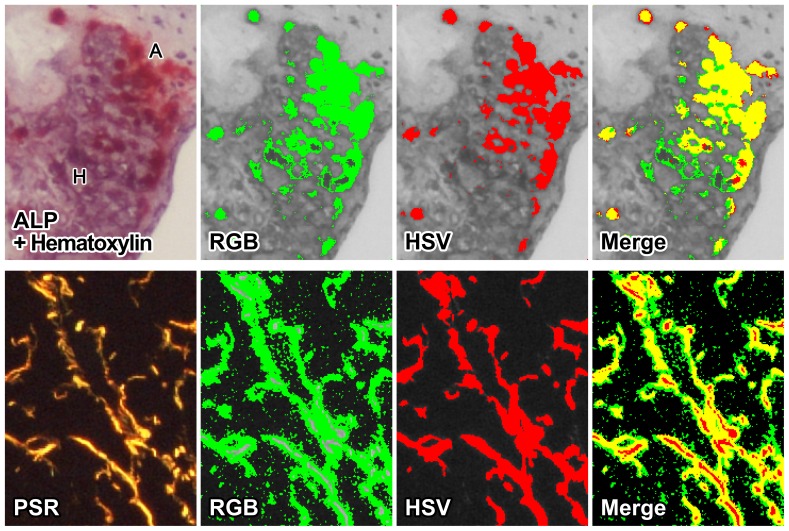
*CSH* is a powerful tool for a variety of stains. Common histological stains, displaying the fidelity of *CSH*. (Top panels): A mouse aorta stained with alkaline phosphatase (ALP, red) for detection of early calcification, with Gill's hematoxylin as counterstaining (purple), which depicts advanced calcification. ALP stain is scarlet red (denoted “A” in the top left panel), while hematoxylin is a shade of purple (denoted “H” in the top left panel). Visually, the hematoxylin interferes with the ALP, making it difficult to see where the ALP stain begins and ends. We analyzed the section for ALP-positive area using both CSH and an RGB-based method. (Bottom panels): A mouse liver stained with picrosirius red staining visualized using polarized light microscopy for detection of fibrosis. We analyzed the section using both CSH and an RGB-based method. The RGB method was unable to register the brightest parts of the stain as positive (gray), and falsely interpreted stain artifacts as positive areas (green in both “Merge” images).

*CSH* remains powerful and accurate across a variety of histological stains, including some stains that have traditionally been difficult to interpret quantitatively. Figure 5 (top panels) shows the power of *CSH* when analyzing a mouse aortic arch stained with alkaline phosphatase (ALP, red), despite similarity in appearance between positive (ALP) and negative (Hematoxilyn, purple) plaque areas. Figure 5 (bottom panels) also shows the fidelity with which *CSH* can accurately detect a high contrast signal provided by picrosirius red-positive collagen staining. Green shows overestimated results on both “Merge” images.

## Discussion

The human eye can detect color thanks to three types of cone cells in the retina, each of which absorbs light most readily at a specific wavelength: long-wavelength (λ = 575 nm), middle-wavelength (λ = 535 nm), and short wavelength (λ = 430 nm) cones. By analyzing the strength of signal received from each type of cone cell, the brain can infer the intensity spectrum of incident light. In theory, all colors that can be interpreted by humans can be represented faithfully by describing the intensity of long, middle, and short-wavelength light. However, physical and technical limitations currently disallow a perfect representation of color as interpreted by the brain.

A “color space” is any mathematical representation of perceived colors. The RGB (red-green-blue) color space is one of the most common color spaces. In RGB space, the intensities of red, green, and blue light are each used to define an axis on a three-dimensional graph. Because the pure colors red, green, and blue are close to the absorbance peaks of the three cone cells of the eye, nearly any visible color can be emulated. Most modern computer monitors physically represent color in RGB space: each pixel of a computer screen emits narrowband light at three frequencies – red, green, and blue. Due to the trichromatic nature of monitors, RGB space is the most common method for digitally storing color data [7]. There are four other basic color spaces that are commonly used, each offering advantages and disadvantages for a variety of tasks.

Using the HSV color space during quantitative image analysis is more robust and useful than performing similar analyses using an unaltered RGB color space. Our program, *CSH*, which makes use of the HSV color space, returned significantly more robust analyses across a range of section thicknesses than the RGB method, and was visibly more consistent. Using HSV thresholding instead of RGB enabled *CSH* to identify necrotic tissues in MI mouse hearts; necrotic tissue identification would be impossible in the RGB space, and would have to be performed manually by tracing the infarction to eliminate it from the ROI.

A handful of commercially available image analysis programs allow users to select a binary thresholding method, including options for RGB, HSV, Hue Saturation Lightness (HSL), or other, less common color spaces. However, these programs do not typically offer intuitive and descriptive toolbars to allow thresholding parameters to be set manually the way *CSH* does, and many of them do not allow for as many simultaneously applied thresholds. *CSH* has the added benefit of being available online for free as a web application. Analysis is fast, reliable, and easy.

We have not tested *CSH* on images larger than 10240×7680 (78 million) pixels, or file sizes larger than 225MB. However, there is no theoretical limit to the file size of images *CSH* can process, but, as with any image analysis program, exceptionally large images, such as those produced by slide scanners, may take a long time to process. There are circumstances where an HSV analysis will offer no benefits over a classic RGB analysis. In single immunofluorescence staining, colors are often pure, falling into clearly distinct groups in RGB space as well as in HSV. However, in the case of multiple fluorescent stains, and when a stain may be detected in multiple color channels, *CSH* may offer more nuanced analyses compared to RGB methods.

In conclusion, we have presented a new fast and reliable method for histological image analysis. Our program, *Color Selection HSV*, can be used as an analytical tool by researchers and clinical pathologists to quantify histological images. It remains accurate despite variability in sample thickness and stain saturation, making it more reliable than other common image analysis methods and programs.

## Supporting Information

Figure S1 Creating an image binary. RGB analysis method, used as an example to demonstrate how thresholding parameters work. In most cases, histological stains make use of bright, noticeable colors that emphasize the target area to be easily visualized. However, the stain color is not all or nothing. There is always a gradient of color expression due to the variation in density of a stain's target. This means that we need to be able detect a range of color elements, and recognize slightly varied colors as positive area. The left panel in the figure shows a theoretical tiny pixel-array (3×3 = 9 pixels) in an image. Each pixel has a unique set of RGB values, each of which is some shade of purple. The central panel shows the thresholding-range set for each of the three color-elements. This means that for any pixel in the array to be considered positive, it must express a red color-element (R) at an intensity between 180 and 240, a green color-element (G) intensity between 0 and 50, and a blue color-element (B) intensity between 160 and 200. The right panel shows the result after thresholding. Only if all three conditions are met is a pixel considered to be positive area. This process is repeated for all pixels in a sample image, and may be repeated up to three times using three different thresholding parameter sets if we need to extract three different colors from an image.
(TIF)

Figure S2 Setting threshold parameters. The appearance of our software tools for color separation by the HSV thresholding method (upper) and by the RGB thresholding method (bottom). Three thresholding parameters in each color space can be seen above the sample image in each panel.
(TIF)

Figure S3 The effect of image resolution on analysis. Part of 2×- and 10×-Images were cut out to be able to show same area that a 20×-image covers. To conveniently compare each image, the 2×-images (RGB method, MT and HSV method) were enlarged 10 times and the 10×-images were enlarged 2 times in length. The percentages of area extraction were normalized so that the measured areas in the 2×-images would represent 100%. The error bars show standard deviations from 3 different sections from different mouse heart samples. The percent area extraction shows an increase in measured muscle area and a decrease in measured collagen area when the RGB method was performed at higher magnifications. However, the HSV method only induced slight increase in collagen area even when the method analyzed higher-magnification images.
(TIF)

Figure S4 The effect of image downsizing on analysis. Left Panel: An original, 3000×3000 pixel image (MT) was downsized serially by half (in each dimension) down to 1/16 size (188×188 pixel). Each downsized image was analyzed by the RGB method and the HSV method using constant parameters. Right Panel: The percent areas of muscle and collagen extracted by both methods were normalized to the values of original image. The HSV method was more consistent than the RGB method, as it showed only a 5% deviation from the original image values, even when analyzing an image 1/16 of the original size.
(TIF)

Figure S5 The effect of artifacts on analysis. We examined the place where the section was folded (the darker area located at the center in the top left panel). The illustration (bottom right panel) shows how the section was folded, and we recognized that there were three patterns: 1) the collagen area (C) was folded to 2 layers, indicated as C+C; 2) the muscle area (M) was folded to 3 layers, indicated as M+M+M; 3) one collagen area, one muscle area and the mixed area were folded to 3 layers, indicated as C+C/M+M. The RGB method showed only negative space at the fold. This means that the RGB analysis was unable to identify any positive tissue at the site of the fold. However, CSH showed very consistent results in the all three patterns. Only the red color was observed in the area specified with “M+M+M”, and the blue color was seen at the area specified with “C+C”. In the mixed area (C+C/M+M), both red and blue are observed.
(TIF)

Figure S6 The effect of focal plane on analysis. To test the consistency of CSH when analyzing defocused images, we prepared serial defocused images by tilting the slide glass (θ = 6°). This made 100 µm height difference in focal points between upper side and bottom side of the image (10× objective lens, 2560×1920 pixels image size). We took 6 serial out-of-focus pictures, focusing on points every 384 pixels along the direction of the slope. The frequencies of area extraction of the muscle and the collagen were normalized to the average measured muscle and collagen areas. CSH showed more consistent results than those of the RGB method.
(TIF)

Methods S1 Color space conversion. Equation 1, max(*R*, *G*, *B*), returns the maximum value (*MAX*) of the three RGB color elements (R; red, G; green, B; blue). Similarly, equation 2, min(*R*, *G*, *B*), returns the minimum (*MIN*) value of the three color elements, R, G, or B. Hue (*H*) is then determined by one of the four options, equation 3A – eq. 3D, as determined by which of the three color elements has the greatest value. Based on these equations, it is possible for *H* to be between 0 and 360. The saturation (*S*) and the value (*V*) are determined by the equations 4 and 5. They range from 0 to 255. For convenience, we show here some examples of the actual color conversion process (Table S1).
(DOC)

Table S1 Examples showing actual color conversion from the RGB colors to the HSV colors. In order to deepen the understanding how the actual color conversion from the RGB colors to the HSV colors is performed we provided Table S1. Each RGB color has unique set of red (R), green (G) and blue (B) ranging from 0 to 255 (8-bit). From the equations (eq. 1, 2) in Methods S1 the values of MAX, MIN and Option are obtained. Through the calculation using equations eq. 3 – 5 (eq. 3 is specified with the Option) hue (H), saturation (S) and value (V) are obtained. For example the first color “*Red*” on the list uniquely having a RGB color-element set of {R: 255, G: 0, B: 0} is converting to a HSV color-element set of {H: 0, S: 255, V: 255}. It should be noticed that violet colors show same hue no matter if they are pale or dark (compare colors numbered 6 to 8, they have same hue = 300).
(TIF)
