# Supplementary figures and images for: A Novel Quantitative Approach for Eliminating Sample-To-Sample Variation Using a Hue Saturation Value Analysis Program
Source: PLoS One. 2014 Mar 3;9(3):e89627. doi: 10.1371/journal.pone.0089627 (PMC3940696; doi:10.1371/journal.pone.0089627)

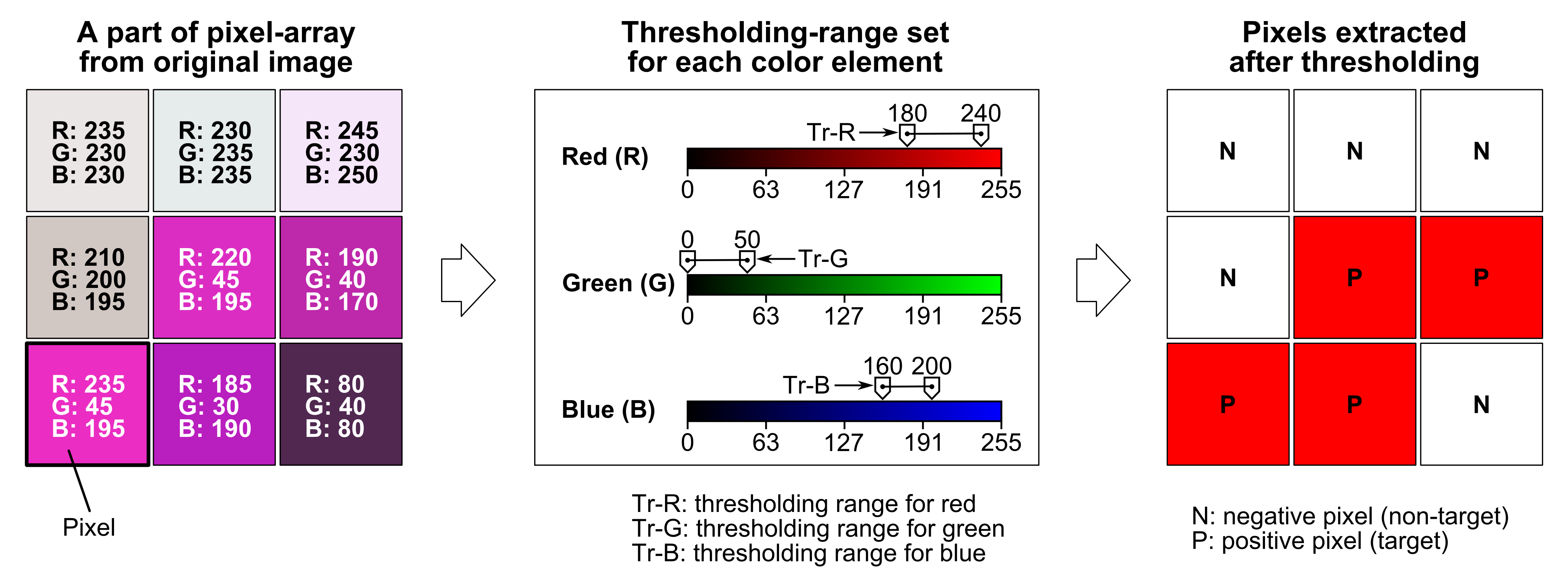

Supplement: Figure S1 — Creating an image binary. RGB analysis method, used as an example to demonstrate how thresholding parameters work. In most cases, histological stains make use of bright, noticeable colors that emphasize the target area to be easily visualized. However, the stain color is not all or nothing. There is always a gradient of color expression due to the variation in density of a stain's target. This means that we need to be able detect a range of color elements, and recognize slightly varied colors as positive area. The left panel in the figure shows a theoretical tiny pixel-array (3×3 = 9 pixels) in an image. Each pixel has a unique set of RGB values, each of which is some shade of purple. The central panel shows the thresholding-range set for each of the three color-elements. This means that for any pixel in the array to be considered positive, it must express a red color-element (R) at an intensity between 180 and 240, a green color-element (G) intensity between 0 and 50, and a blue color-element (B) intensity between 160 and 200. The right panel shows the result after thresholding. Only if all three conditions are met is a pixel considered to be positive area. This process is repeated for all pixels in a sample image, and may be repeated up to three times using three different thresholding parameter sets if we need to extract three different colors from an image. (TIF) [file pone.0089627.s001.tif]

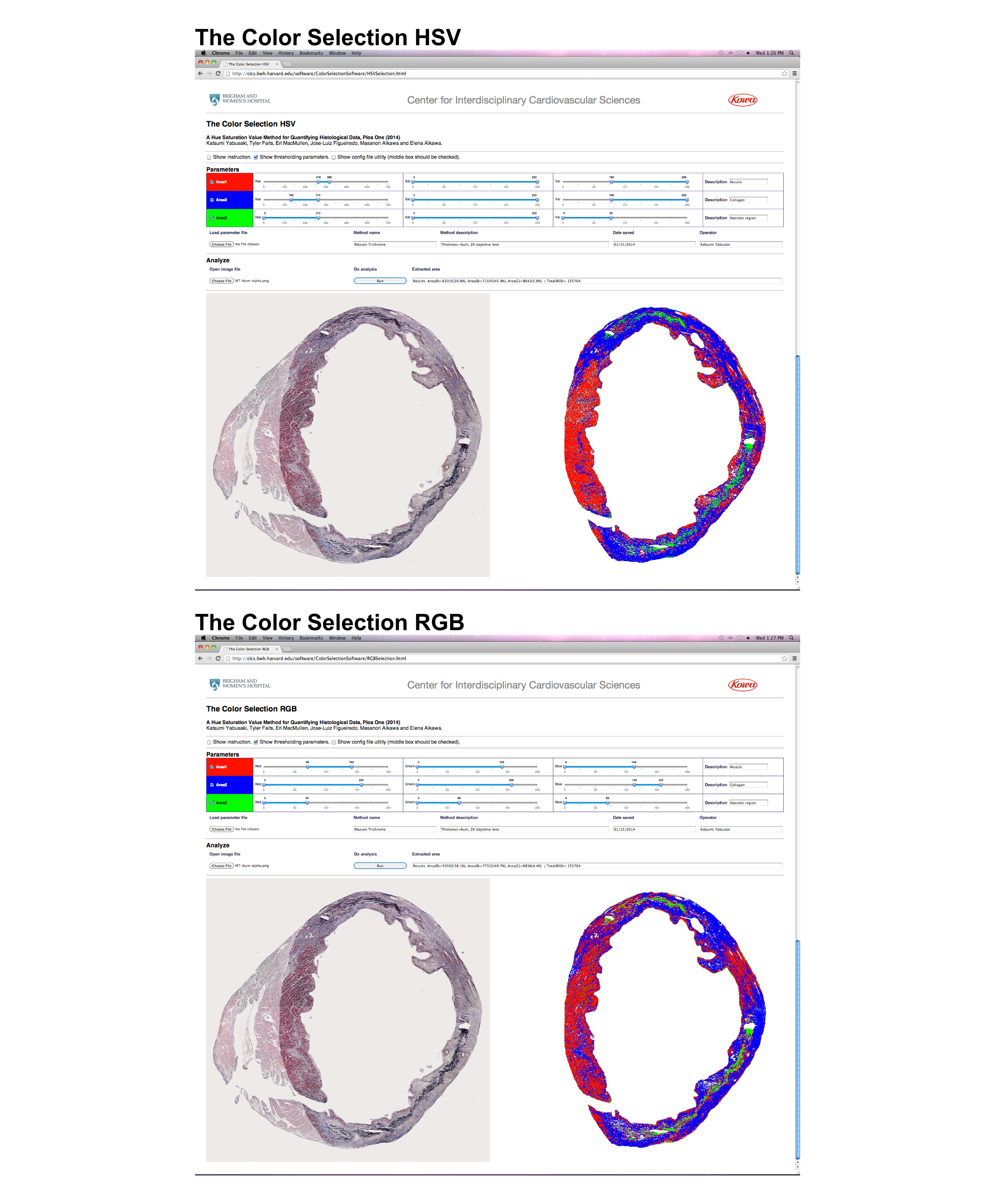

Supplement: Figure S2 — Setting threshold parameters. The appearance of our software tools for color separation by the HSV thresholding method (upper) and by the RGB thresholding method (bottom). Three thresholding parameters in each color space can be seen above the sample image in each panel. (TIF) [file pone.0089627.s002.tif]

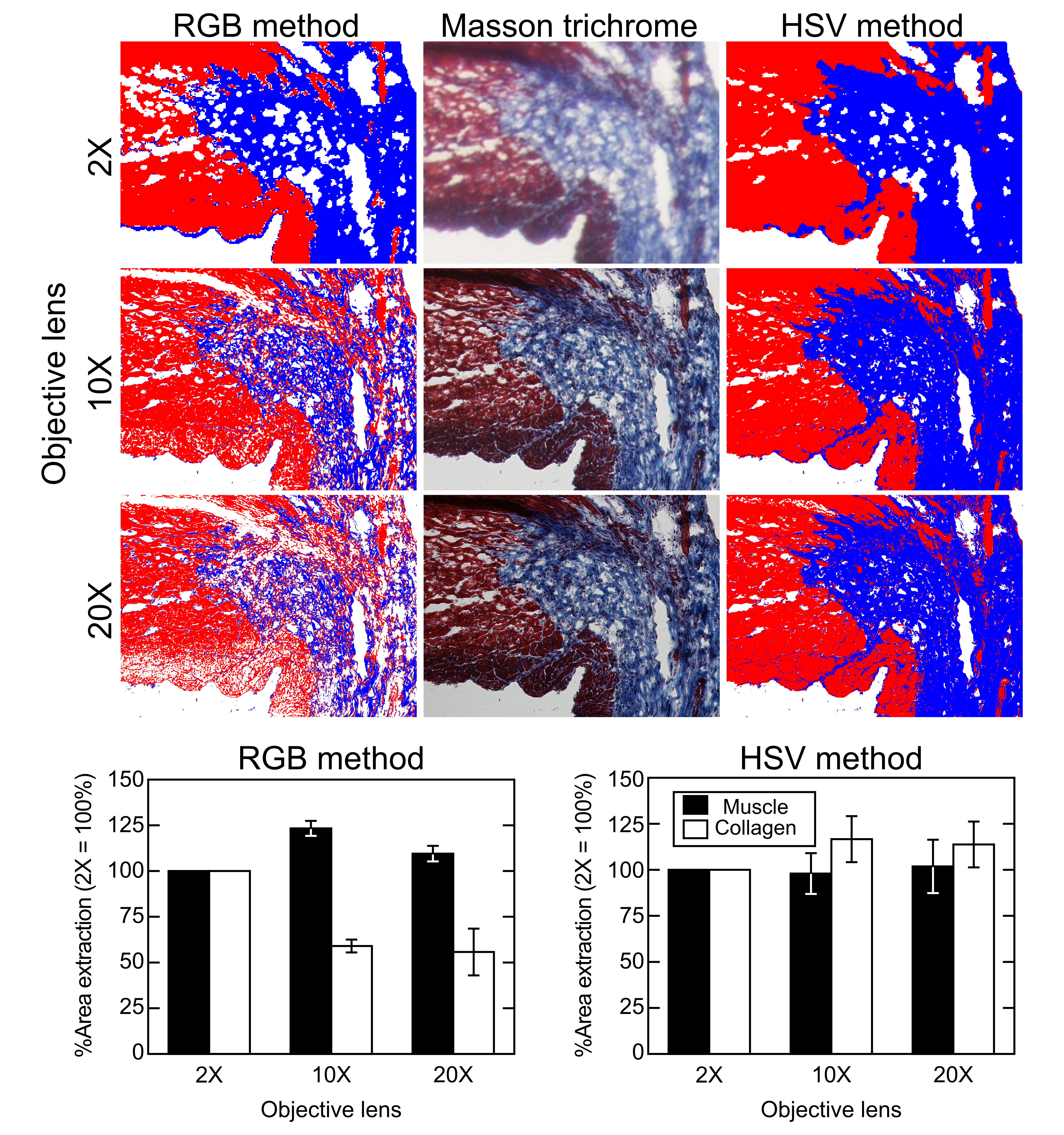

Supplement: Figure S3 — The effect of image resolution on analysis. Part of 2×- and 10×-Images were cut out to be able to show same area that a 20×-image covers. To conveniently compare each image, the 2×-images (RGB method, MT and HSV method) were enlarged 10 times and the 10×-images were enlarged 2 times in length. The percentages of area extraction were normalized so that the measured areas in the 2×-images would represent 100%. The error bars show standard deviations from 3 different sections from different mouse heart samples. The percent area extraction shows an increase in measured muscle area and a decrease in measured collagen area when the RGB method was performed at higher magnifications. However, the HSV method only induced slight increase in collagen area even when the method analyzed higher-magnification images. (TIF) [file pone.0089627.s003.tif]

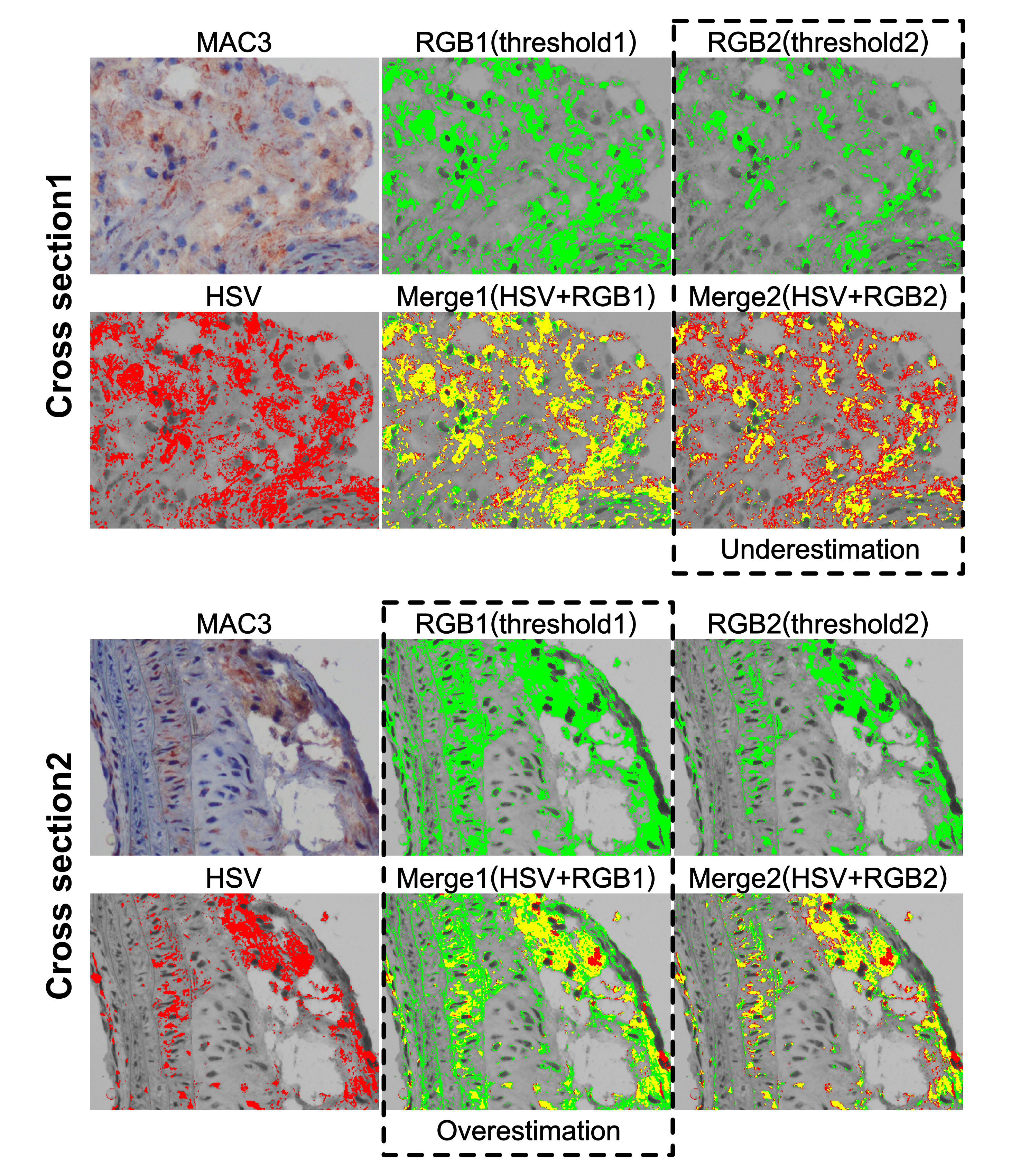

Supplement: Figure S4 — The effect of image downsizing on analysis. Left Panel: An original, 3000×3000 pixel image (MT) was downsized serially by half (in each dimension) down to 1/16 size (188×188 pixel). Each downsized image was analyzed by the RGB method and the HSV method using constant parameters. Right Panel: The percent areas of muscle and collagen extracted by both methods were normalized to the values of original image. The HSV method was more consistent than the RGB method, as it showed only a 5% deviation from the original image values, even when analyzing an image 1/16 of the original size. (TIF) [file pone.0089627.s004.tif]

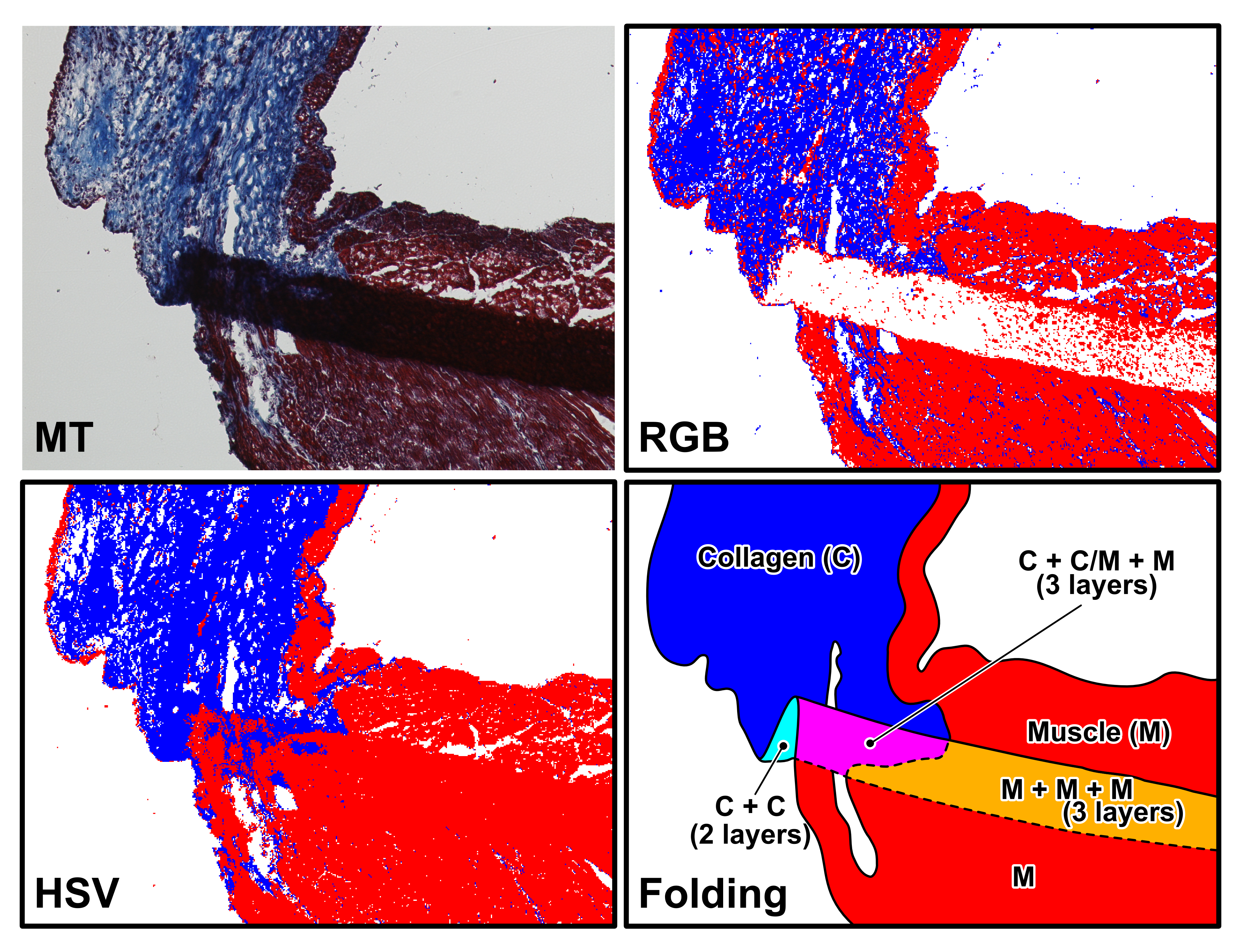

Supplement: Figure S5 — The effect of artifacts on analysis. We examined the place where the section was folded (the darker area located at the center in the top left panel). The illustration (bottom right panel) shows how the section was folded, and we recognized that there were three patterns: 1) the collagen area (C) was folded to 2 layers, indicated as C+C; 2) the muscle area (M) was folded to 3 layers, indicated as M+M+M; 3) one collagen area, one muscle area and the mixed area were folded to 3 layers, indicated as C+C/M+M. The RGB method showed only negative space at the fold. This means that the RGB analysis was unable to identify any positive tissue at the site of the fold. However, CSH showed very consistent results in the all three patterns. Only the red color was observed in the area specified with “M+M+M”, and the blue color was seen at the area specified with “C+C”. In the mixed area (C+C/M+M), both red and blue are observed. (TIF) [file pone.0089627.s005.tif]

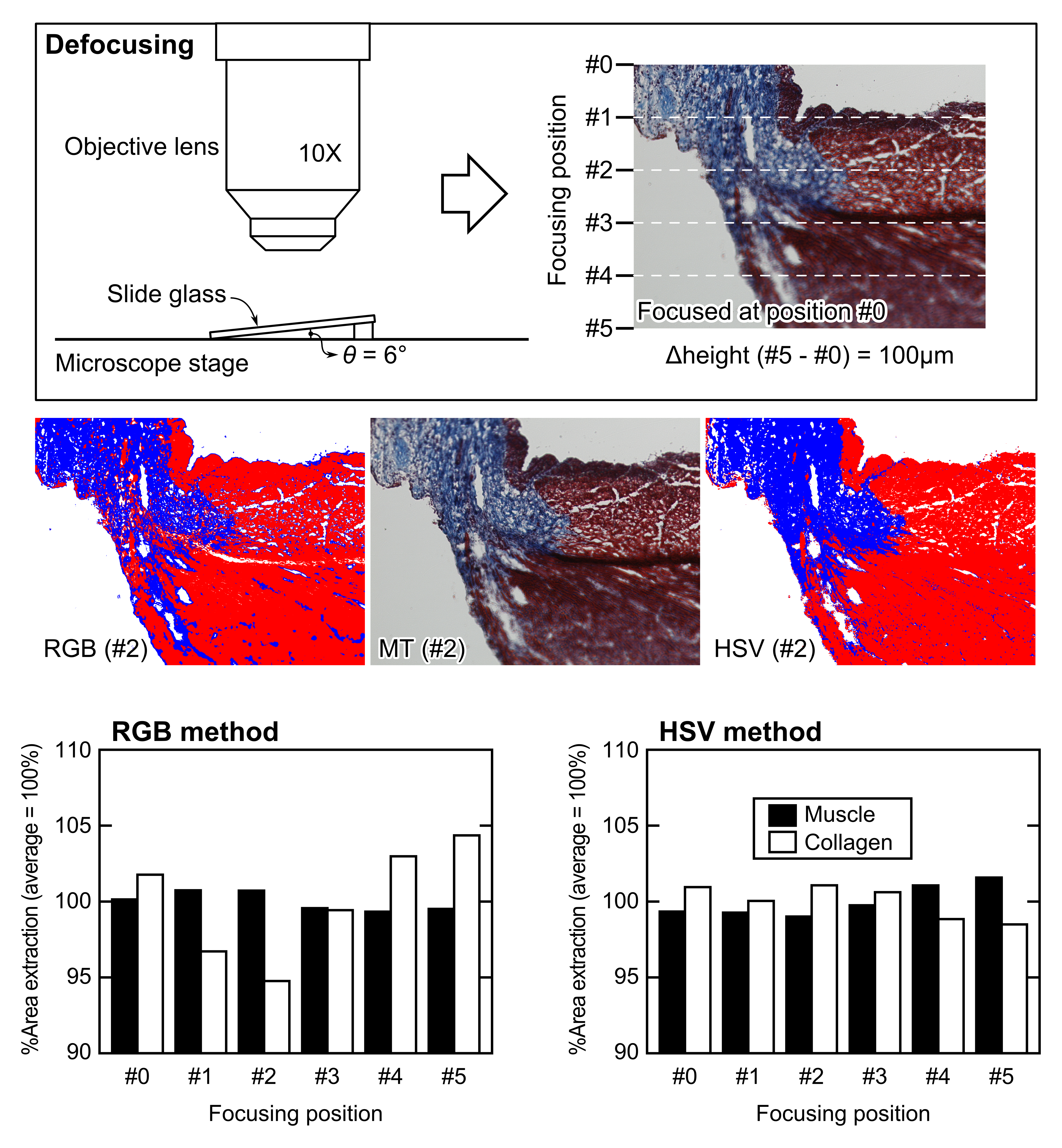

Supplement: Figure S6 — The effect of focal plane on analysis. To test the consistency of CSH when analyzing defocused images, we prepared serial defocused images by tilting the slide glass (θ = 6°). This made 100 µm height difference in focal points between upper side and bottom side of the image (10× objective lens, 2560×1920 pixels image size). We took 6 serial out-of-focus pictures, focusing on points every 384 pixels along the direction of the slope. The frequencies of area extraction of the muscle and the collagen were normalized to the average measured muscle and collagen areas. CSH showed more consistent results than those of the RGB method. (TIF) [file pone.0089627.s006.tif]
