# Supplementary material for: A Novel Quantitative Approach for Eliminating Sample-To-Sample Variation Using a Hue Saturation Value Analysis Program
Source: PLoS One. 2014 Mar 3;9(3):e89627. doi: 10.1371/journal.pone.0089627 (PMC3940696; doi:10.1371/journal.pone.0089627)
Supplement: Methods S1 — Color space conversion. Equation 1, max(R, G, B), returns the maximum value (MAX) of the three RGB color elements (R; red, G; green, B; blue). Similarly, equation 2, min(R, G, B), returns the minimum (MIN) value of the three color elements, R, G, or B. Hue (H) is then determined by one of the four options, equation 3A – eq. 3D, as determined by which of the three color elements has the greatest value. Based on these equations, it is possible for H to be between 0 and 360. The saturation (S) and the value (V) are determined by the equations 4 and 5. They range from 0 to 255. For convenience, we show here some examples of the actual color conversion process (Table S1). (DOC) [file pone.0089627.s007.doc]

**Supplemental Methods**

**S7. Color Space Conversion**


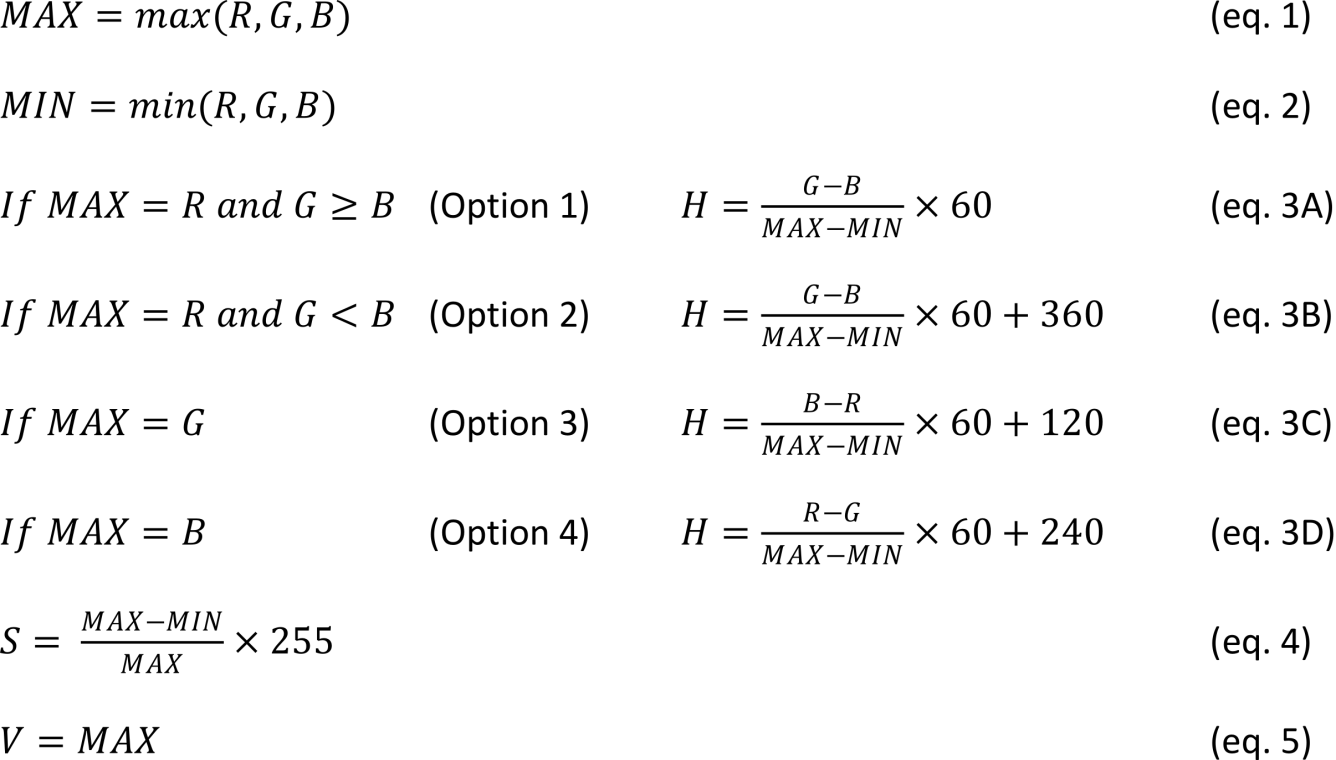
In some cases, digital cameras may have a larger bit depth for the color elements (such as 10 bits or 14 bits for each color element). However, we usually use an 8-bit depth value (0 - 255) for each color element. This means the RGB color space has 24 bits color depth (8 bits x 3 = 24 bits). The color conversion from the RGB color space to the HSV color space using 24-bit color depth was performed according to the equations 1 to 5;
